# Effects of Annealing and Heat-Moisture Treatment on Structural Characterization and In Vitro Digestibility of Debranched Mung Bean Starch

**DOI:** 10.3390/foods15132281

**Published:** 2026-06-25

**Authors:** Yifei Lu, Xinyu Wang, Lujing Xu, Cong Teng, Jin Feng, Li Cui, Xindi Hu, Kaiyang Ma, Zhi Chai, Ying Li

**Affiliations:** 1 Institute of Agro-Product Processing, Jiangsu Academy of Agricultural Sciences, Nanjing 210014, China; luyifei3377@163.com (Y.L.); 15996295085@163.com (L.X.); tengcong95@163.com (C.T.); fengjinzju@163.com (J.F.); clisu1@163.com (L.C.); 20210057@jaas.ac.cn (X.H.); makaiyang110@163.com (K.M.); sophia_chai@163.com (Z.C.); 2 School of Food and Biological Engineering, Jiangsu University, Zhenjiang 212013, China; wangxinyu522623@163.com

**Keywords:** mung bean starch, processing methods, multi-scale structure, digestibility

## Abstract

Resistant starch type 3 (RS3) exhibits physiological benefits in regulating post-meal blood sugar levels and enhancing gut microbiota balance. In this study, mung bean starch was isolated and modified through debranching, annealing (ANN) and heat-moisture treatment (HMT). The multi-scale structures investigated by SEM, FT-IR, and XRD unveiled the formation of short-range ordered, helix, and crystalline structures. Notably, RS3 formed through debranching and HMT exhibited both a remarkably high RS content of 54.71% and a low estimated glycemic index (eGI) of 51.78. Statistical evaluation through correlation and stepwise regression analyses suggested that short-range molecular order was the primary factor associated with the resistance of RS3 to enzymatic hydrolysis, while the chain length of B-chains exerted secondary yet notable influences. This work provided novel insights into the interplay between processing methodologies, ordered molecular structures, and starch digestibility resistance.

## 1. Introduction

Dietary carbohydrates are predominantly supplied by starch, a key macronutrient in human nutrition. Starch fractions can be categorized according to their enzymatic digestibility profiles, comprising the following three distinct types: resistant starch (RS), slowly digestible (SDS) and rapidly digestible (RDS) [1]. RS offers positive health benefits to consumers, encompassing the prevention of colon cancer, blood glucose control, cholesterol reduction, and a decreased incidence of obesity, among others. This is attributed to its resistance to digestion in the small intestine and subsequent fermentation by gut microbiota in the large intestine, leading to the production of short-chain fatty acids [2,3]. RS is categorized into five distinct subtypes, designated RS1 through RS5. RS3, in particular, has become a major focus of investigation because it demonstrates remarkable thermal resistance and is recognized as safe for dietary intake [4]. RS3 belongs to the category of retrograded starch, also known as recrystallized RS, which is acquired via the retrogradation process of amylose, or alternatively, through the recrystallization and structural rearrangement of debranched starch molecules [5,6,7]. Hence, the proportion of amylose stands as a key determinant in the generation process of RS3.

Mung bean (*Vigna radiata*), a legume, has been extensively cultivated in southeast Asia, particularly Vietnam, for millennia. It contains 26–31% starch and is a primary source of RS in human diets [8,9]. Mung bean starch (MS) is characterized by a substantial proportion of amylose, along with notable granular stability, resistance to shear forces when in paste form, and restricted swelling capacity. Due to the exceptional transparency and strong gel-forming ability of its starch paste, MS is considered the optimal choice among raw materials for the production of starch noodles. However, due to its high amylose content, MS demonstrates significant syneresis and exhibits poor storage stability [10,11].

Currently, the most commonly employed method for increasing RS3 content is enzymatic debranching of starch (utilizing enzymes such as isoamylase and pullulanase), which induces the recombination of short linear glucans. Additionally, numerous researchers have explored various approaches to enhance the RS content in starch. After 24 h of debranching and recrystallization, the RS contents of cassava starch and potato starch reached 35% and 48%, respectively, which were higher than those of their native counterparts [12]. After debranching waxy corn starch with pullulanase and subsequent recrystallization at 25 °C for 24 h, the RS content reached 70.7% [13]. Heat-moisture treatment (HMT) and annealing (ANN) represent two thermo-mechanical processes for modifying starch. These techniques effectively alter the functional properties of starch granules while preserving their structural integrity and have been extensively employed to enhance the RS content of MS. HMT is conducted at elevated temperatures above 90 °C under restricted moisture levels (less than 35%), whereas ANN is carried out at temperatures below the gelatinization point in the presence of excess moisture (greater than 40%) [14]. Both HMT and ANN induce significant modifications to the physicochemical characteristics of starch. These alterations are evidenced by a marked decrease in swelling capacity and amylose release, concurrent with elevated gelatinization temperatures, enhanced resistance to thermal breakdown, and a notable retardation of retrogradation [15]. In addition, both HMT and ANN can enhance the yield of RS [2]. After treatment with HMT and ANN, the contents of RS and SDS in corn, pea and lentil starches increased significantly, while the content of RDS decreased sharply [16]. The formation of RS during HMT and ANN is attributed to the interactions between starch chains. Among these, amylose is considered the most crucial factor influencing the strength of starch gels, as it interacts with lipids to form helical complexes and/or with amylopectin to establish a robust gel network, thereby conferring resistance to enzymatic hydrolysis [17].

Despite these advances, gaps still remain. Most studies have focused on either debranching alone or HMT/ANN alone, and the combined effects of debranching followed by HMT or ANN on RS3 formation in MS have not been systematically investigated. Second, the relationship between multi-scale structural alterations and the resulting digestibility of recrystallized MS remains poorly understood. Consequently, the primary aim of this study was to investigate the alterations in multi-scale structure and digestibility of pullulanase-debranched MS following HMT and ANN treatments. The results yielded by this study have the potential to expedite the development of innovative food components that function as RS/resistant dextrin, offering enhanced health-promoting effects.

## 2. Materials and Methods

### 2.1. Materials

Mung bean cultivars were commercially sourced from online suppliers. Pepsin and pancreatin were obtained from Sigma-Aldrich Trading Company (Shanghai, China). Porcine pancreas α-amylase, glycosylase and DNS reagent were purchased from Yuanye Biotecnology (Shanghai, China). Pullulanase was from Macklin Reagent Biotecnology (Shanghai, China). Amylose and Starch Assay Kits were purchased from Solarbio Biotecnology (Beijing, China).

### 2.2. Starch Extraction

A hydration process was initiated by combining mung beans with distilled water in a beaker. The blend was formulated utilizing a volumetric ratio of 1:3 (bean to water), followed by a 12 h equilibration period at ambient temperature. The soaked beans were subsequently disrupted using an Ultra-Turrax T18 high-speed disperser (IKA Works Guangzhou, Guangzhou, China) running at 6000 rpm for 2 min with a solid-to-liquid ratio of 1:10 (*w*/*v*). The slurry was passed through a 200-mesh sieve to remove mung bean fiber. The sieved slurry was then extracted with 0.1 M NaOH (4 g/1000 mL) at a ratio of 1:15 (*w*/*v*) for 3 h. After extraction, the supernatant was discarded, and the precipitate was washed repeatedly with distilled water until the supernatant became colorless and pH-neutral. The residue was then centrifuged at 3000× *g* for 10 min and the upper impurities were scraped off. The harvested pellet sample underwent drying in an oven set at 45 °C, was subsequently passed through an 80-mesh screen, and then preserved at −20 °C pending further analysis. This sample was denoted as MS.

### 2.3. Preparation of Resistant Starch from Mung Bean Starch

The debranching process of MS was carried out in accordance with the method outlined by Huong et al. [18] using pullulanase. Mung bean starch (10 g) was mixed with acetate buffer (0.02 M, pH 5.6) at a starch-to-buffer ratio of 1:15 (*w*/*v*). Initially, the mixture underwent a heat treatment process by being placed in a water bath set at a temperature of 100 °C for a duration of 10 min. After cooling the cooked slurry to 55 °C, pullulanase (30 U/g starch) was incorporated to initiate enzymatic digestion, which proceeded for 16 h at this temperature with constant agitation. Enzymatic activity was terminated by mixing the solution with an equivalent volume of 95% ethanol (1:1 ratio), subjected to centrifugation at a force of 3000× *g* for 15 min. The supernatant was decanted and the residue was washed twice with distilled water. The debranched starch was then dried in a ventilated oven at 45 °C for 24 h until the moisture content reached 10–11%. The dried debranched starch was stored in airtight glass containers until further analysis. This sample was denoted as DS.

The DS sample underwent HMT [19]. In a 250 mL Erlenmeyer flask, starch was adjusted to 30% moisture content (*w*/*w*). The flask was then securely sealed and allowed to equilibrate at ambient temperature for a duration of 24 h. Following this equilibration period, the sample underwent heating in an oven set at 110 °C for 8 h. After heating, the sample was retrieved through centrifugation at a force of 10,000× *g* for 30 min and subsequently dried overnight at a temperature of 40 °C. This sample was denoted as DHS.

The debranched starch sample underwent ANN [19]. DS was blended with distilled water, maintaining a solid-to-liquid proportion of 1:2 (*w*/*v*), transferred into an Erlenmeyer flask, sealed and maintained at 45 °C for 24 h. The mixture was subsequently separated via 10,000× *g* centrifugation for 30 min and then dried at 45 °C overnight. This sample was denoted as DAS.

### 2.4. Starch Digestibility

Starch digestion analysis in vitro was conducted following the Englyst methodology. A 200 mg sample aliquot was combined with 15 mL of 0.2 M sodium acetate buffer (pH 5.2), along with 4 mL of an enzymatic solution comprising α-amylase (290 U/mL) and amyloglucosidase (15 U/mL). The resulting mixture was subsequently incubated at 37 °C under continuous agitation. Aliquots of the hydrolysate (0.5 mL) were collected at predetermined intervals (0, 20, 30, 60, 90, 120, and 180 min). Each aliquot was immediately mixed with 4.5 mL of ethanol to quench the reaction. After centrifugation, the glucose concentration was quantified utilizing the dinitrosalicylic acid (DNS) method, as outlined by Miller in 1959 [20]. Additionally, the concentrations of RDS, SDS and RS were assessed:

(1) RDS (%) = (G_20_ − FG) × 0.9/TS

(2) SDS (%) = (G_120_ − G_20_) × 0.9/TS

(3) RS (%) = [TS − (RDS + SDS)]/TS

where TS denoted the total starch content; FG designated the initial glucose concentration; and G_20_ and G_120_ corresponded to glucose concentrations measured at 20 and 120 min, respectively.

The hydrolysis index (HI) was determined by calculating the ratio between the area beneath the hydrolysis curve of the sample and that of the reference material (fresh white bread). Subsequently, the estimated glycemic index (eGI) was computed as follows:

(4) GI = 39.71 + 0.549 × HI

### 2.5. Chain Length Distribution

Starch chain length profiling was conducted using a methodology adapted from the work of Chang et al. [21]. Accurately 2 mg of starch was weighed into a 2 mL centrifuge tube, 1.0 mL of ultrapure water was added, and boiled in a water bath until completely dissolved. A total of 10 μL of sodium acetate buffer solution (1 M, pH = 4.5) and 1.5 μL of isoamylase solution were added and maintained a constant temperature at 40 °C for more than 4 h for starch digestion. After the reaction was terminated, the pH was adjusted to 7 with NaOH (0.1 M) and centrifuged at 8000 r/min for 30 min. The distribution of amylopectin chain length was determined using an ICS-6000DC high-pressure ion chromatography system (Thermo Fisher Scientific, Waltham, MA, USA).

### 2.6. Determination of Morphology of Starches

The microstructures of MS, DS, DHS and DAS complexes were observed using a ZEISS EVO-LS10 scanning electron microscope (SEM) (Carl Zeiss AG, Oberkochen, Germany). Samples were affixed to silicon plates and underwent a gold sputtering process prior to observation [22].

### 2.7. Determination of Crystalline Structure of Starches

X-ray diffraction (XRD) analysis was performed using a D2 PHASER X-ray diffractometer (Bruker AXS, Karlsruhe, Germany) to examine the crystalline properties of both the native and modified MS specimens. The experimental setup utilized Cu-Kα radiation, operating under conditions of 40 kV voltage and 40 mA current, while diffraction data were captured within a 2θ angular span from 5° to 30°. Crystal structure and crystallinity were assessed via deconvolution of diffraction peaks and calculation of peak area ratios [23].

### 2.8. Determination of Short-Range Ordered Structure of Starches

FT-IR spectral data were recorded using a Nicolet iS50 Fourier transform infrared spectrometer (equipped with an attenuated total reflectance (ATR) accessory) (Thermo Fisher Scientific, Waltham, MA, USA). The wavelength coverage extended from 4000 cm^−1^ to 500 cm^−1^, with a spectral resolution set at 4 cm^−1^. The structural parameters DO (degree of molecular order) and DD (degree of double helix) were calculated using the intensity ratios of 1047/1022 cm^−1^ and 995/1022 cm^−1^, correspondingly, according to the method of Ma et al. [24].

### 2.9. Solubility and Swelling Power

Starch solubility and swelling capacity were evaluated using a procedure adapted from Li et al., with slight adjustments [25]. The starch suspension (1%) was heated in a water bath at 55 °C, 65 °C, 75 °C, 85 °C and 95 °C, respectively, for 30 min, and then cooled to room temperature. After centrifugation at 3000× *g* for 10 min, the supernatant was discarded. The supernatant and precipitate were separated, followed by the evaporation of a fraction of the supernatant at 110 °C. Subsequently, the swollen starch precipitate was measured for its weight. The solubility and swelling capacity were then determined using the following formulas:

(5) $$S\%=\frac{A}{B}\times 100\%$$

(6) $$P\%=\frac{B}{W\times (1-S)}\times 100\%$$

where

S denoted solubility;

A represented the mass of the dried supernatant (g);

W was dried starch weight (g);

P indicated the swelling capacity;

B corresponded to the mass of the sediment (g).

### 2.10. Thermogravimetric Analysis (TGA)

An accurately weighed sample (10 mg) was placed in a testing crucible, and the measurement was conducted using SDT650 TGA (TA Instruments, New Castle, DE, USA) under a nitrogen atmosphere serving as the protective gas. The nitrogen flux was kept constant at 50 mL/min, while the sample underwent analysis across a temperature span from 10 °C to 800 °C, with a heating increment of 10 °C per minute.

### 2.11. Statistical Analysis

Each experiment was conducted with three replicates, and findings are presented as mean ± SD. One-way analysis of variance (ANOVA) was utilized for comparing groups, with a significance threshold set at *p* < 0.05. Mean comparisons were conducted using Duncan’s multiple range test at the 0.05 significance level. Additionally, correlation and stepwise regression analyses were performed using GraphPad Prism 9.0 software.

## 3. Results and Discussion

### 3.1. Moisture Content and the Proportion of Starch-Related Components in Starch Samples

Table 1 presented the determined contents of total starch, amylose, amylopectin, and moisture in the starch samples. All sample groups maintained total starch contents exceeding 90%. DS, DHS and DAS had similar levels of amylose and amylopectin with the range from 65.69% to 66.25% and 33.75% to 34.31%, respectively. In stark contrast, the amylose content of MS, which was without debranching treatment, was merely 27.36%. The mung bean variety used in this experiment was classified as medium-amylose starches in the study by Nguyen et al. [26]. The findings indicated that the incorporation of pullulan enzyme yielded a notable positive impact on the hydrolysis of amylopectin. As a debranching enzyme, pullulanase functions by cleaving the α-1,6 glycosidic bonds within amylopectin, thereby generating a substantial quantity of amylose. This amylose subsequently aggregates and organizes into densely packed structures, ultimately forming RS that exhibits resistance to digestion [27]. In this study, the amylose content following three different processing methods served as evidence for this phenomenon.

### 3.2. In Vitro Digestibility of Starch Samples

The values of SDS, RDS and RS of MS, DS, DHS and DAS were presented in Table 1. The MS from the mung bean variety selected for this experiment named “Tailai” contained a remarkably high RS content of 32.3%, which was markedly higher than that documented in comparable studies [18]. This indicated that this variety possessed natural resistance to enzymatic hydrolysis. Compared with MS, the concentration of RDS in recrystallized MS significantly decreased by 15.4–33.6% while the concentration of RS in recrystallized MS significantly increased by 18.9–69.5%. The increase in RS content of recrystallized MS was attributed to the formation of a higher number of double-helical structures by the linear chains of amylopectin, which exhibited higher crystallinity and a more ordered molecular structure. Shi et al. [13] demonstrated that following debranching and recrystallization processes, the RS content and crystallinity of waxy corn starch notably rose, owing to the formation of highly crystalline structures within the recrystallized starch aggregates. The reasons for the formation of this part will be discussed later.

The estimated glycemic index (eGI) serves as a straightforward and cost-effective approach, wherein the digestion rate coefficient is contingent upon both the type of model employed in the research and the concentration of enzymes within the system [28]. It is believed that starch digestion is initially slow due to inaccessibility of enzyme which later gelatinizes by cooking and permeability extends to the whole matrix making starch digestion faster [29]. The production of glucose and its derivatives within the human body fluctuates depending on various factors, such as cooking techniques, age, physical activity levels, and overall health status. Nevertheless, this particular method boasts an accuracy rate of up to 95% in distinguishing between low and high glycemic index (GI) foods [30]. As indicated in the table, after processing, the eGI values of recrystallized MS significantly decreased. The eGI values of DS, DHS, and DAS dropped from 62.02 in the unprocessed MS to 58.94, 51.78, and 56.78, respectively. Among them, DHS exhibited the lowest value, indicating that the combination of debranching and HMT can significantly reduce the eGI of MS, thereby mitigating the impact of starch on postprandial blood glucose levels. Meanwhile, RS is currently recognized as an important component that influences the GI of rice [31]. This finding aligned with our experimental outcomes, revealing that DHS, possessing the highest RS content, demonstrated the lowest eGI value.

### 3.3. Multi-Scale Structures of Starch Samples

#### 3.3.1. Chain Length Distributions

**Figure 1:**
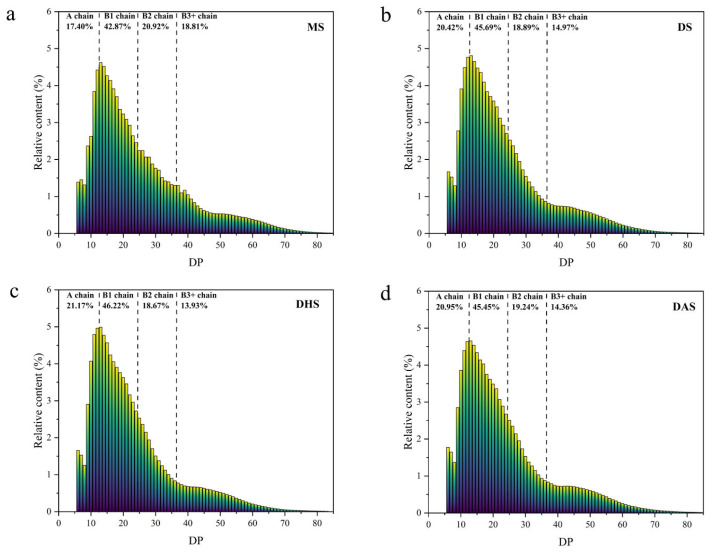
Chain length distributions of (**a**) MS, (**b**) DS, (**c**) DHS and (**d**) DAS.

The primary structure of branched starch consists of glucose units linked by α-1,4 glycosidic bonds, while α-1,6 glycosidic bonds attach side branches to the main chain, forming branching points. By employing pullulanase for hydrolysis, chain segments of the branching part with different degrees of polymerization (DPs) can be generated. This enables a comprehensive examination of the structural characteristics of branched starch. Figure 1 presented the ion chromatograms depicting the chain-length distributions of all samples analyzed using Thermo ICS-6000DC (Thermo Fisher Scientific, Waltham, MA, USA). Starch-based amylopectin can be categorized into the following four chain classifications: A (DP 6–12), B1 (DP 13–24), B2 (DP 25–36), and B3+ (DP ≥ 37) [32]. Table 2 also presented the calculated average chain length (ACL) values for MS, DS, and recrystallized MS samples. Our study revealed a notable reduction in the ACL value of the modified MS following treatment. Notably, DHS displayed the minimal ACL value, comprising 22.58 glucosyl residues. After debranching treatment, the B3+ chains all decreased significantly. DHS demonstrated the largest proportion of A-chains, accounting for 21.17%, mainly due to the breakdown of B3+ chains. This indicated that short branched chains were predominant in DHS.

By hydrolyzing amylopectin’s α-1,6 bonds, pullulanase treatment enhances the relative proportion of linear starch chains [33]. The observed difference in chain length ratio between DHS and DAS may be attributed to the effects of HMT and ANN treatments, as these two processes could potentially lead to interactions among short starch chains during the recrystallization process. In other words, during the recrystallization process, the short and linear chains generated by debranching may engage with each other, with the primary molecular interactions shifting from amylose-amylopectin to amylose-short linear chain interactions [34]. Thermal decomposition of amylopectin molecules was detected during initial heating phases. For instance, Lu et al. [35] found that the macromolecular fraction in rice starch diminished throughout HMT procedure. Ayenampudi et al. [36] reported an increase in the proportion of B2 chains during ANN treatment, indicating that ANN can alter the chain length distribution of starch molecules. These results aligned with the outcomes of our experiments. The high proportions of A-chains and B1-chains in DHS suggested that more short chains are formed during the HMT process following debranching. Previous experiments have demonstrated that the DP13–24 chain segment, namely the B1-chains, served as a crucial structural basis for the formation of RS3 [37]. This also elucidated the reason for the high RS content in DHS.

#### 3.3.2. Scanning Electron Microscope (SEM)

**Figure 2:**
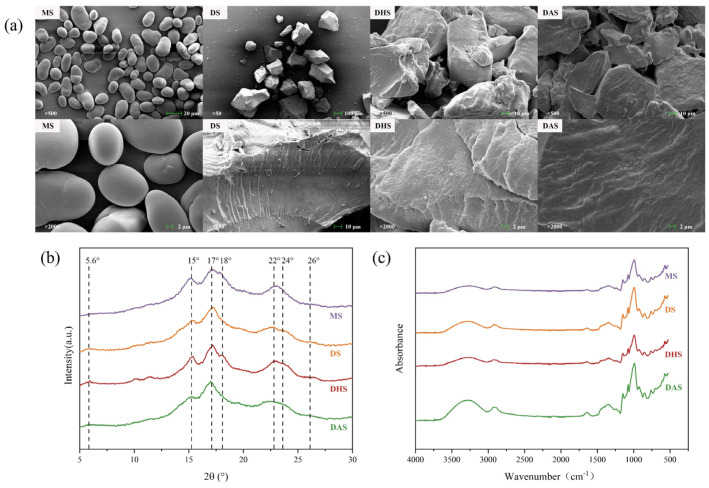
SEM images (**a**), XRD patterns (**b**), and FT-IR spectra (**c**) of MS, DS, DHS, and DAS.

The photographs in micromorphology of the mixed systems captured through SEM were displayed in Figure 2a. The native MS exhibited a bimodal distribution in terms of granular shape and size. Specifically, the larger granules displayed an oval or elliptical morphology, while the smaller ones were characterized by a round shape [26]. All the processed recrystallized mung bean starches exhibited irregular, block-like aggregates with sharp edges and surface features adorned with scaly fragments. These characteristics differed significantly from the morphological structure of native MS, indicating that debranching had disrupted the molecular organization of amylopectin. Debranching broke down starch-protein molecules to generate short-chain linear starch molecules, which were then aggregated during the retrogradation process to form crystals within debranched starch granules [12,24]. Our outcome was similar to findings from previous studies [18]. Moreover, the recrystallized starch displayed a considerably greater size in comparison to the native mung bean starch. This was likely because, during the gelatinization process, water molecules infiltrated the inner regions of starch granules, leading to the leaching of amylose. Subsequently, during the retrogradation process, the leached amylose rearranged to form aggregates.

#### 3.3.3. Crystalline Structure of Starches

The XRD patterns of MS, DS, DHS, DAS and DMS were revealed in Figure 2b. MS manifested a distinctive A-type crystalline configuration, corroborating the earlier observations made by Ohwada et al. [38]. The principal diffraction peaks in the A-type X-ray pattern of MS were observed at 2θ angles of 15.16°, 17.02°, 17.80°, and 23.12°. Following the treatments, DS, DHS, and DAS all formed new structures with distinct X-ray diffraction patterns with peaks at 5.6° 2θ, 15° 2θ, 17° 2θ, 18° 2θ, 23° 2θ, 24° 2θ and 26° 2θ, displaying a typical type C crystalline structure. MS lost its original granular integrity. Unlike all previously reported cases where the crystal structure of starch transformed from type A or type C to type B after debranching [12,13,39], in this study, the DS changed from type A to type C, which might be attributed to the reorganization of double helices at low temperatures [40]. The current results also indicated that HMT and ANN treatments did not alter the crystal structure of DS. Although DHS and DAS generated more short chains after treatment, they did not change the relative crystalline region in a way that would lead to a modification of the crystal structure. This observation paralleled the research outcomes reported by Nguyen et al., [18] wherein the XRD patterns of MS remained unchanged following treatment with a combination of citric acid, HMT, or ANN.

Following debranching, the native starch’s amylopectin crystalline structure undergoes disruption, releasing linear glucan chains which are the hydrolytic products of pullulanase treatment. During the rapid retrogradation process, these linear glucan chains initially form single-helical structures, resulting in a lower relative crystallinity (RC) compared to that of native starch (Table 2). After recrystallization, the RC values increased, with the RC of DHS reaching 34.31%, which was higher than that of the original MS. This is because the external chains of amylopectin (A chains and B1 chains) wind around each other in the form of double helices, forming ordered regions or the “crystalline lamellae” within starch granules [41]. Since enzymatic hydrolysis typically occurred in the amorphous regions of starch, the formation of more starch recrystallized regions can enhance the digestive resistance of recrystallized RS [42].

#### 3.3.4. Short-Range Ordered Structure of Starches

The short-range ordered structures of MS, DS, DHS, and DAS, as determined by FT-IR spectroscopy, are presented in Figure 2c. FT-IR spectral data covering 500–4000 cm^−1^ were collected to analyze helical conformations and double-helix arrangements, following established spectral interpretation methods [43]. The frequency intervals spanning 3500–3000 cm^−1^ and 2970–2850 cm^−1^ correspond to the stretching vibrations of the hydrogen-bonded O–H group and the asymmetric stretching vibrations of CH_2_, respectively, as reported in reference [44]. Meanwhile, the peak at 1649 cm^−1^ indicates the stretching vibrations of the C–O or C–C bonds, according to reference [45]. Indeed, the absorbance peaks observed at 1047 cm^−1^, 1022 cm^−1^, and 995 cm^−1^ serve as the principal characteristic indicators in FT-IR spectroscopy. The positions of the characteristic peaks in the processed starch samples did not change significantly, indicating that neither debranching, HMT, nor ANN treatment altered the functional groups of MS. This suggested that these processing methods solely involved rearrangement among starch molecular chains or modifications in intermolecular hydrogen bonding.

The intensity ratios at 1047/1022 cm^−1^ and 995/1022 cm^−1^ were employed to calculate DO and DD, serving as indicators of the short-range ordering within starch granules and the internal alterations in the double-helix structure of starch, respectively [39]. Under debranching treatment, DO and DD values for DS, DHS, and DAS were notably higher than those of MS. This indicated that the linear short-chain molecules in DS exhibited a highly ordered arrangement. After recrystallization, DHS and DAS developed more ordered and periodic amorphous-crystalline structures, thereby facilitating the formation of domains containing moderately densely packed starch chains. Among them, DHS exhibited the highest DO and DD values. This phenomenon was ascribed to the generation of numerous short linear chains, which augmented the mobility of molecular chains and facilitated the alignment of double helices. Such well-structured and densely packed double-helical configurations likely render starch more resistant to enzymatic digestion [46]. These results align with our observations from in vitro digestion and XRD assessments.

### 3.4. TG and DTG and Solubility and Swelling Power

**Figure 3:**
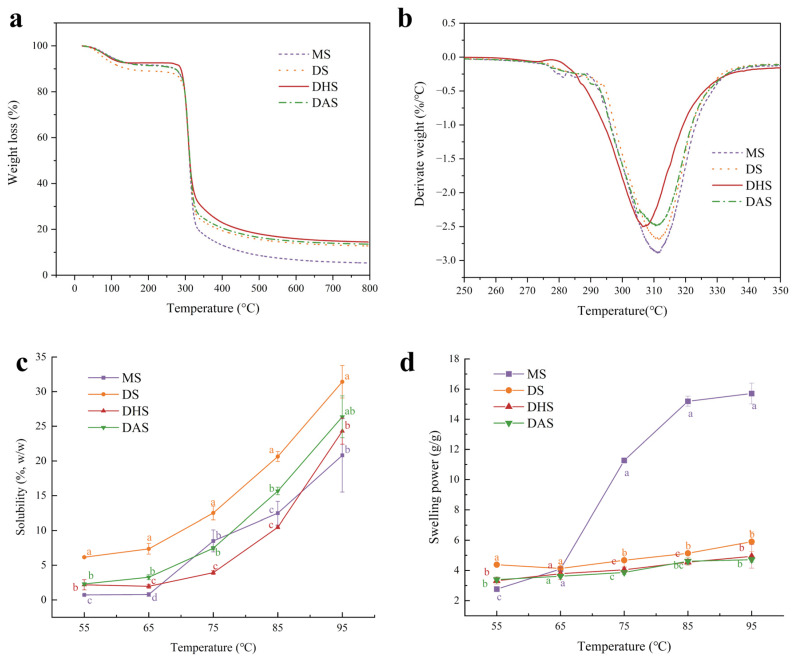
TG (**a**), DTG (**b**), solubility (**c**) and swelling power (**d**) profiles of MS, DS, DHS, and DAS.Different lowercase letters indicate significant differences.

During the thermogravimetric testing process, the evaporation of moisture and the thermal decomposition of the sample often occur simultaneously. The weight loss (TG) and derivate weight (DTG) of samples were illustrated in Figure 3a and Figure 3b, respectively. It could be obviously observed that TG of mung bean samples was generally divided into three stages. In the initial stage, TG primarily occurred within the temperature range of 30 °C to 150 °C, mainly resulting from the evaporation of both free and some bound water. In the subsequent stage, TG predominantly happened between 150 °C and 320 °C, owing to the depolymerization and decomposition reactions of starches, along with the release of water and other volatile products. At elevated temperatures, the macromolecules dissociated and the spatial structure gradually collapsed, resulting in a rapid rate of weight loss. The TG in the third stage mainly occurred between 320 °C and 800 °C, with a relatively slow rate of weight loss during this period [46,47]. In addition, at 600 °C, the weight loss rates of DS, DHS, and DAS were approximately 80%, which was significantly lower than that of MS alone. This indicated that DS exhibited greater thermal stability compared to MS, and also indirectly suggested that the maximum thermal degradation rate (MTDR) and degradation rate of RS were both inferior to those observed in native starch [47]. Compared with MS, the thermal decomposition peak temperature (Td) of DHS (306.49 ± 0.30 °C) and DAS (310.95 ± 0.08 °C) decreased, while that of DS (311.54 ± 0.11 °C) remained similar. The maximum thermal degradation rate (MTDR) decreased progressively from 2.90% (MS) to 2.69% (DS), 2.50% (DHS), and 2.49% (DAS). The enhanced thermal stability could likely stem from strengthened molecular interactions among starch chains and the amorphous crystalline structure of starch. Additionally, the disintegration of the structural crystals might require additional thermal energy.

The solubility of starch serves as an indicator of the capacity of amylose molecules to elute from starch granules at a given temperature. Notably, both solubility and swelling power are intricately linked to the extent of interaction among starch chains within the crystalline and non-crystalline domains of starch granules [48]. The solubility and swelling power of starch samples were shown in Figure 3c,d. The solubility and swelling power of each sample group demonstrated an ascending trend as the temperature rose. This occurrence might be ascribed to the weakening of interactions between starch molecules brought about by the temperature increase. Consequently, the structure of the starch granules was disrupted, leading to the release of short-chain molecules and a subsequent increase in the quantity of starch dissolved in water. Simultaneously, owing to alterations in the starch particle architecture, water molecules were able to penetrate the interior of the starch and engage in interactions with the starch molecules [49]. With the temperature elevation, the swelling capacity of each sample group gradually increased, with notable differences starting to emerge at 75 °C. As illustrated in the figure, recrystallized starches displayed notably lower swelling capacity compared to MS, suggesting their reduced tendency for water absorption and expansion. Similarly, with a rise in temperature, the solubility of each sample group also gradually improved, with significant differences beginning to appear at 85 °C. The figure revealed that recrystallized starch exhibited significantly reduced solubility compared to MS, making it less soluble in water. This reduction in solubility was likely attributed to the formation of a more compact and ordered crystalline structure in recrystallized starches. Specifically, debranching generated abundant short linear glucan chains (A and B1 chains), which subsequently re-associated into dense double helices and crystalline lamellae during retrogradation and hydrothermal treatments. The increased short-range molecular order (DO and DD) and higher RC limited water penetration and starch chain dissolution, thereby decreasing solubility.

### 3.5. Correlation and Stepwise Regression Analyses Between Starch Multiscale Structures and Digestion Resistance

**Figure 4:**
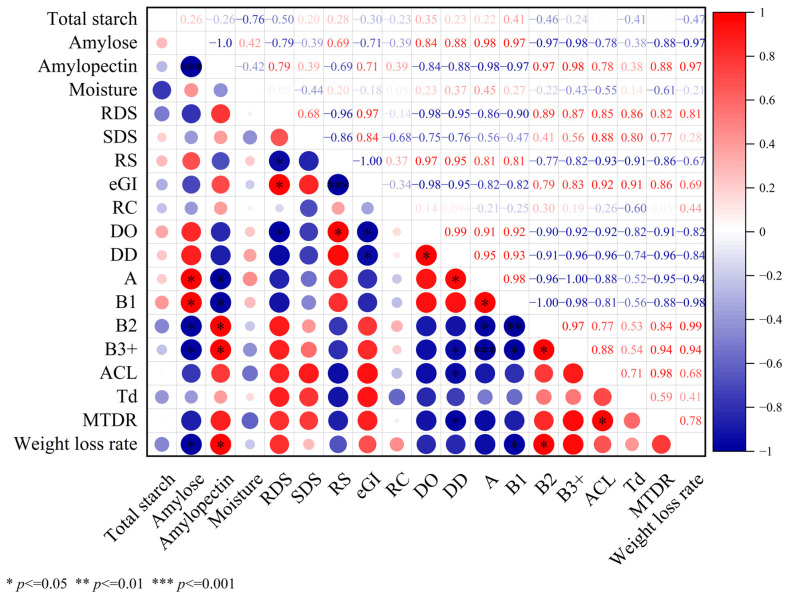
Pearson correlation analysis of multi-scale structures and in vitro digestibility of MS, DS, DHS and DAS.

The multi-scale architectures of starch serve as critical determinants influencing enzyme binding and catalytic efficiency. Initially, Pearson correlation analysis disclosed the link between digestive characteristics and multi-scale structures in recrystallized mung bean starches. Figure 4 illustrated significant positive correlations between RS levels and multiple parameters as follows: amylose (R = 0.69), RC (R = 0.37), DO (R = 0.97), DD (R = 0.95), A (R = 0.81), and B1 (R = 0.81). Conversely, RDS and eGI displayed inverse relationships with these structural features. Studies have demonstrated that short-range ordered structures can effectively impede enzyme diffusion, thereby enhancing digestion resistance [50,51]. Notably, long-range ordered structures were found to exhibit superior inhibitory effects on starch digestibility [52]. Significantly, the eGI value demonstrated a strong correlation with RS and ordered structural features (*p* < 0.01). Collectively, the digestibility resistance of recrystallized RS was governed by its hierarchical structural organization, wherein ordered molecular arrangements played a dominant role while densely packed aggregates contributed substantially to the overall effect.

To pinpoint the pivotal structural features that inhibit starch enzymatic digestion, stepwise regression analysis was utilized. Following the elimination of collinear variables, the most suitable regression models were constructed (Table 3). The levels of RDS, RS, and eGI were predominantly influenced by DO, with respective beta coefficients of −0.984, 0.973, and −1.465. This suggested that DO value, that is, short-range orderliness, was the key characteristic responsible for digestion resistance. Overall, the short-range ordered structure appeared to be the most influential factor associated with the digestion resistance of recrystallized RS, while the chain length of B-chains exerted secondary yet notable influences, as suggested by the correlation and stepwise regression analyses.

## 4. Conclusions

In this study, the thermal stability, solubility, swelling power, chain length, multi-scale structures and digestibility of recrystallized RS formed under different processing methods were clarified. The innovative integration of debranching with HMT and ANN markedly improved the functional properties and in vitro digestibility profiles of MS. Modified starches demonstrated substantially enhanced RS levels along with improved eGI values relative to native starch counterparts. Notably, DHS exhibited both a remarkably high RS content of 54.71% and a low eGI of 51.78. Among these factors, the short-range ordered structure was identified as the most prominent parameter correlated with digestion resistance. However, this study was conducted only at laboratory scale, further validation in animal models and clinical trials is needed to confirm the physiological benefits, and optimization of processing parameters for commercial manufacturing is required. Despite these limitations, the modified mung bean starches with high RS content and optimal physicochemical properties show great potential as functional food ingredients.

## Figures and Tables

**Table 1:** The total starch, amylose, amylopectin and moisture content of starch samples.

**Samples**	**Total Starch (%)**	**Amylose (%)**	**Amylopectin (%)**	**Moisture (%)**
MS	90.76 ± 0.16 ^b^	27.36 ± 0.38 ^b^	72.64 ± 0.38 ^a^	7.57 ± 0.22 ^b^
DS	91.92 ± 0.32 ^a^	66.15 ± 0.07 ^a^	33.85 ± 0.07 ^b^	7.66 ± 0.28 ^b^
DHS	91.79 ± 0.71 ^a^	65.69 ± 0.48 ^a^	34.31 ± 0.48 ^b^	7.72 ± 0.54 ^b^
DAS	90.03 ± 0.07 ^b^	66.25 ± 0.57 ^a^	33.75 ± 0.57 ^b^	8.90 ± 0.68 ^a^
**Samples**	**RDS (%)**	**SDS (%)**	**RS (%)**	**eGI**
MS	47.45 ± 0.36 ^a^	20.27 ± 1.07 ^b^	32.28 ± 0.71 ^d^	62.02 ± 0.48 ^a^
DS	39.63 ± 0.08 ^b^	21.99 ± 0.86 ^a^	38.38 ± 0.89 ^c^	58.94 ± 0.32 ^b^
DHS	31.50 ± 0.43 ^c^	14.09 ± 0.43 ^d^	54.71 ± 0.45 ^a^	51.78 ± 0.34 ^d^
DAS	40.13 ± 0.82 ^b^	16.00 ± 0.91 ^c^	43.88 ± 0.73 ^b^	56.78 ± 0.36 ^c^

Values are expressed as mean ± standard deviation (SD), *n* = 3. Values in the same column with different superscript are significantly different (*p* < 0.05).

**Table 2:** Thermal characteristic parameters, RC, DO, DD, ACL, of starch samples.

Parameter	Samples			
	MS	DS	DHS	DAS
T_d_ (°C)	311.58 ± 0.11 ^a^	311.54 ± 0.11 ^a^	306.49 ± 0.30 ^c^	310.95 ± 0.08 ^b^
MTDR (%)	2.90 ± 0.05 ^a^	2.69 ± 0.01 ^b^	2.50 ± 0.05 ^c^	2.49 ± 0.01 ^c^
Weight loss rate (%)	94.66 ± 0.00 ^a^	84.84 ± 0.00 ^d^	85.52 ± 0.00 ^c^	87.22 ± 0.00 ^b^
Crystallization type	A	C	C	C
RC (%)	32.80 ± 0.95 ^a^	23.64 ± 0.39 ^c^	34.31 ± 1.21 ^a^	29.73 ± 1.45 ^b^
DO (1047/1022 cm^−1^)	1.054 ± 0.009 ^c^	1.066 ± 0.003 ^bc^	1.079 ± 0.001 ^a^	1.069 ± 0.001 ^ab^
DD (995/1022 cm^−1^)	1.044 ± 0.009 ^c^	1.062 ± 0.009 ^b^	1.079 ± 0.001 ^a^	1.070 ± 0.001 ^ab^
A chain (%)	17.40 ± 0.02 ^d^	20.42 ± 0.03 ^c^	21.17 ± 0.03 ^a^	20.95 ± 0.03 ^b^
B1 chain (%)	42.87 ± 0.05 ^d^	45.69 ± 0.03 ^b^	46.22 ± 0.03 ^a^	45.45 ± 0.07 ^c^
B2 chain (%)	20.92 ± 0.02 ^a^	18.89 ± 0.03 ^c^	18.67 ± 0.03 ^d^	19.24 ± 0.03 ^b^
B3+ chain (%)	18.81 ± 0.02 ^a^	14.97 ± 0.03 ^b^	13.93 ± 0.03 ^d^	14.36 ± 0.03 ^c^
Average chain length (ACL)	23.19 ± 0.019 ^a^	22.99 ± 0.005 ^b^	22.58 ± 0.001 ^d^	22.65 ± 0.003 ^c^

Values in the same column with different superscript are significantly different (*p* < 0.05).

**Table 3:** The stepwise regression analysis between structural factors and in vitro digestibility (RDS, RS, eGI) of MS, DS, DHS and DAS.

Dependent Variable	Item	B	Beta	t	*p*
RDS	Constant	704.774	/	8.399	0.014
	DO	−623.333	−0.984	−7.926	0.016
RS	Constant	−918.189	/	−5.683	0.030
	DO	900.189	0.973	5.945	0.027
eGI	Constant	642.918	/	161.475	0.004
	DO	−612.928	−1.465	−121.570	0.005
	B1	1.519	0.527	43.710	0.015

## Data Availability

The original contributions presented in this study are included in the article. Further inquiries can be directed to the corresponding author.
